# The Intensity of Hospital Care Utilization by Dutch Patients With Lung or Colorectal Cancer in their Final Months of Life

**DOI:** 10.1177/1073274819846574

**Published:** 2019-06-03

**Authors:** Yvonne de Man, Femke Atsma, Mariska G. Oosterveld-Vlug, Linda Brom, Bregje D. Onwuteaka-Philipsen, Gert P. Westert, A. Stef Groenewoud

**Affiliations:** 1 Radboud University Medical Center, Radboud Institute for Health Sciences, Scientific Center for Quality of Healthcare, Nijmegen, the Netherlands; 2 Department of Public and Occupational Health, Amsterdam Public Health Research Institute, Expertise Center for Palliatie Care, Amsterdam UMC, Vrije Universiteit Amsterdam, the Netherlands; 3 IKNL, Netherlands Comprehensive Cancer Organization, Utrecht, the Netherlands

**Keywords:** colorectal cancer, lung cancer, hospital care, end-of-life, undertreatment, overtreatment

## Abstract

Understanding the overuse and underuse of health-care services in the end-of-life (EoL) phase for patients with lung cancer (LC) and colorectal cancer (CRC) is important, but knowledge is limited. To help identify inappropriate care, we present the health-care utilization profiles for hospital care at the EoL of patients with LC (N = 25 553) and CRC (N = 14 911) in the Netherlands between 2013 and 2015. An administrative database containing all in-hospital health-care activities was analyzed to investigate the association between the number of days patients spent in the emergency department (ED) or intensive care unit (ICU) and their exposure to chemotherapy or radiotherapy. Fewer patients received hospital care as death neared, but their intensity of care increased. In the last month of life, the average numbers of hospital bed days, ICU days, and ER contacts were 9.0, 5.5, and 1.2 for patients with CRC, and 8.9, 6.2 and 1.2 for patients with LC in 2015. On the other hand, the occurrence of palliative consultations ranged from 1% to 4%. Patients receiving chemotherapy 6 months before death spent fewer days in ICU than those who did not receive this treatment (odds ratios: CRC = 0.6 [95% confidence interval: 0.4-0.8] and LC = 0.7 [0.5-0.9]), while those receiving chemotherapy 1 month before death had more ED visits (odds ratios: CRC = 17.2 [11.8-25.0] and LC = 15.8 [12.0-20.9]). Our results showed that patients who were still receiving hospital care when death was near had a high intensity of care, yet palliative consultations were low. Receiving chemotherapy or radiotherapy in the final month of life was significantly associated with more ED and ICU contacts in patients with LC.

## Introduction

Lung cancer (LC) and colorectal cancer (CRC) are 2 of the 5 most common types of cancer in the Netherlands,^1^ with 12 600 and 15 000 new patients, respectively, diagnosed in 2015. With 5-year survival rates of 18% (LC) and 58% (CRC), survival is low.^1^ Furthermore, patients with LC and CRC have the second and third highest hospital care costs of all patients with cancer.^2^

Due to screening programs, more patients with cancer are identified at earlier disease stages. Cancer is increasingly becoming a chronic disease as a result of new chemotherapies and targeted therapies, meaning that the end-of-life (EoL) phase and the process of dying are longer and more gradual. The early detection of the palliative phase, advance care planning (ACP), and well-considered EoL care are therefore becoming increasingly important; however, we know that medical care can be both overused and underused.^3^ It can be overused in patients with cancer who receive aggressive treatments during the EoL phase, which increases their burden while decreasing their quality of life.^4,5^ The underuse of health care, for instance not having timely EoL discussions, is associated with higher costs and a lower quality of death and dying.^6^

**Key Points**

**Questions**

What is the hospital health-care utilization of Dutch patients with lung cancer (LC) and colorectal cancer (CRC) in the end of life?

**Findings**

We found that, for patients still receiving hospital care when death neared, the intensity of hospital care was high, yet palliative consultations were low. During the last month of life, chemotherapy and radiotherapy were significantly associated with more emergency department and intensive care unit visits in patients with LC.

**Conclusion**

Palliative care is potentially underused in the end-of-life phase. Medical specialists should consider the intensity of treatments when discussing them with patients. Advance care planning can play a substantial role in this.

Analyzing the health-care utilization (HCU) of a patient group forms the first crucial step toward improving their health care and is a tool for the identification of the overuse and underuse of medical care. Here, we aim to present the HCU profiles (HCUP) of patients with LC and CRC using activity-based hospital data. This will provide complementary and contrasting information to the analysis of diagnosis-related group (DRG)–based hospital data, which was previously performed by Bekelman et al.^7^

## Materials and Methods

### Study Population and Selection

This population-based cohort study includes insured Dutch persons (99% of the Dutch population)^8^ who died in 2013, 2014, or 2015 with a diagnosis of LC and/or CRC for whom hospital medical care had been registered. The patients were included as patients with LC if they had been diagnosed with non-small-cell lung carcinoma or small-cell lung cancer, neoplasm bronchus lung, or small- and large-cell bronchus carcinoma. Patients with CRC were included if they had been diagnosed with colorectal malignancy or malignant neoplasm of the colon. Patients under 18 at the time of death or those who lacked a valid citizen service number and post code were excluded from the analysis.

### Data

A national database of administrative hospital data at the health-care activity level was used in the analyses presented here. These activities are registered by all Dutch hospitals (8 academic and 92 general hospitals) and by more than 300 independent treatment centers. The data also contained information regarding the institution providing the care as well as the age and gender of the patients. For 2013 and 2014, the data covered 95% of the delivered and billable care. For 2015, this was approximately 70% due to the administrative delay in registries after care had been given.

### Expert Panel

Five medical experts were involved: 2 pulmonologists/oncologists and 3 experts specializing in internal medicine and oncology. All were specialized in palliative medicine. First, individual in-depth interviews were conducted with 4 experts using an interview guide to gain insights into their current practice and the background and reasons for the HCU patterns of Dutch patients with CRC or LC in the EoL phase. During the analysis phase, the experts classified each health-care activity into meaningful clusters (Table 1) and compared the volume and intensity of EoL HCU to their experiences of daily practice. Also, the experts indicated which types of care had been registered too frequently in their opinion and what would be potential areas for improvement. Finally, the experts argued that unplanned and unwanted admissions such as ED contacts and days in ICU were important to investigate further. They formulated an additional question regarding the association between unwanted admissions and chemotherapy and radiotherapy. It was not possible to identify avoidable or unwanted hospital bed days from the available data; therefore, this study was focused on ICU days and ED contacts.

**Table 1.** Clusters of Health-Care Activities and Descriptions.

Category	Description
Inpatient days	
Hospital bed wlays	A first or subsequent clinical admission, classes A, B, and C
ICU days	Low, medium, and high care in the ICU
Outpatient days	
ED visit	Life support in the ED
Ambulatory visit	A first or return visit
Specialty care consultations	
Palliative care consultation	Specific health-care activity describing a palliative consultation
Multidisciplinary consultation	Co-treatments with other specialists, (clinical) multidisciplinary consultation and activities
Consultation	Consultations between patient and specialist, face-to-face or by telephone
Allied and therapeutic care, per 15 minutes	All allied care and consultations, including physiotherapists, nutritionists, nurse practitioners, psychologists, social workers, optometrists, and geriatric rehabilitation
Diagnostic tests	
Laboratory test	All lab tests regarding hematology and small chemistry
Noninvasive diagnostic test—pathological	Microbiological, histological and pathological (laboratory) tests
Other noninvasive diagnostics	CGA, spirograph tests, interpreting radiology results, preassessments, blood pressure measurement, audiometric tests, and so on
Invasive diagnostics	Includes colonoscopies and bronchoscopies, as well as biopsies and diagnostic punctures
Imaging	
Conventional radiology	Ultrasounds and X-rays, Doppler, duplex, EEG
CT scan	CT scans
MRI scan	MRI scans
PET scan	PET scans
Nuclear scan other than PET	SPECT and radioactive isotope tests
Procedures and treatments	
Chemotherapy	Includes therapies as cisplatin, oxaliplatin, gemcitabin, docetaxel, and irinotecan
CRC/LC surgery	Surgeries registered under diagnosis codes specified in Table 1a and 1b, including tumor resection, tracheotomy, and therapeutic biopsies
Non-CRC/LC surgery	Surgeries other than the aforementioned, for instance, heart transplants; hip replacements, cataract, and hernia surgeries; and resections, reconstructions, and therapeutic biopsies
Biologics	Includes therapies such as bevacizumab, rituximab, and gefitinib
Radiotherapy	Radiation and radiotherapy fractions.
Other (care and items)	Pre- and post-surgery/chemotherapy/radiotherapy care, dental work, devices and implants (pacemaker, Steffeeplate, shunt), prosthetics, dialysis, injections, catheters, and so on

Abbreviations: CGA, comprehensive geriatric assessment; CRC, colorectal cancer; CT, computed tomography; ED, emergency department; EEG, electroencephalogram; ICU, intensive care unit; LC, lung cancer; MRI, magnetic resonance imaging; PET, positron emission tomography; SPECT, single photon computed tomography.

### Clustering Health-Care Activities

More than 13 000 different health-care activities were included in the database, many of which can be grouped together. The experts were therefore asked to define the important and relevant health-care clusters for the EoL phases of patients with CRC and LC separately; it was not necessary to identify the same categories for the 2 illnesses. Subsequently, 2 teams of experts appointed the 75% most frequently occurring health-care activities within each specialism to one of these categories. The process of defining the categories was iterative, meaning that within and between the 2 teams, the categories were adjusted and refined during the clustering process until a consensus was reached. One of the experts checked and finalized the clustering. The health-care activity codes for which no description was available (<1%) and the additional costs for laboratory work, ICU admissions, house visits, or travel expenses were excluded because HCU, not health-care costs, were the focus of this investigation. Also, postmortem examinations were excluded from the clustering as these were not part of the EoL HCU.^9^

### Analyses

The intensity of the HCU was examined by calculating the percentage of patients who received care described within the clusters and determining how often these care activities were performed on average per patient per month in the last 6, 3, and 1 month(s) before death. The associations between radiotherapy or chemotherapy and ICU days or ED contacts were also analyzed using a 2 × 2 frequency table, for which the corresponding odds ratios (OR) and 95% confidence intervals (CI) were calculated. The analyses were performed using SAS Enterprise Guide 9.2.

## Results

### Patient Characteristics

The characteristics of the 25 533 patients with CRC and 14 911 patients with LC included in this study are presented in Table 2. For both CRC and LC, more males than females passed away in the years 2013 to 2015.

**Table 2.** Baseline Characteristics of All Patients with Lung or Colorectal Cancer who Died, 2013 to 2015.

	Lung Cancer, N = 25 533	Colorectal Cancer, N = 14 911
Total		
Gender, %		
Male	15 324 (60.0%)	8494 (56.5%)
Female	10 209 (40%)	6417 (43.5%)
Mean age at death		
Male	70.6	71.6
Female	67.1	72.1

### Health-Care Utilization Profiles

The main clusters of health-care activities for patients with CRC and LC are presented in Table 1, while Table 3 shows the HCUP for these patients by cluster over 3 different time periods: 6 months, 3 months, and 1 month before death. It displays the proportion of patients who received a certain type of care and the average number of times they received it each month. In all 3 years, the 3 most frequently registered health-care activities for patients with both CRC and LC during the EoL phase were ambulatory visits, laboratory tests, and conventional radiology. Overall, the number of patients receiving hospital care decreased as their death neared, although the intensity of care per month increased for those still receiving it.

**Table 3.** Hospital Care Utilization (HCU) Within the Last 6, 3, and 1 Month(s) of Life, the Number of Patients Receiving Certain Types of Care, and the Average Number of Times they Received it.

Colorectal cancer	2013 (N = 5104)									2014 (N = 5683)									2015 (N = 4123)								
Months before death	6			3			1			6			3			1			6			3			1		
	N	%	I^a^	N	%	I	N	%	I	N	%	I	N	%	I	N	%	I	N	%	I	N	%	I	N	%	I
Inpatient days																											
Hospital bed days	3235	63	1.8	2502	49	3.3	1364	27	8.8	3590	63	1.9	2742	48	3.4	1490	26	8.5	2275	55	1.8	1542	37	3.4	780	19	9.0
ICU days	471	9	1.0	379	7	2.0	287	6	5.2	490	9	1.0	374	7	1.9	264	5	5.3	249	6	1.0	175	4	2.0	122	3	5.5
Outpatient days																											
ED visit	2613	51	0.2	1937	38	0.4	977	19	1.2	2924	52	0.2	2121	37	0.4	1097	19	1.2	1784	43	0.2	1121	27	0.4	539	13	1.2
Ambulatory visit	4788	94	0.5	4018	79	0.7	2039	40	1.5	5316	94	0.5	4290	76	0.7	2143	38	1.5	3755	91	0.5	2549	62	0.8	1078	26	1.6
Specialty care consultations																											
Palliative care consultation	201	4	0.3	162	3	0.5	100	2	1.4	266	5	0.3	226	4	0.5	147	3	1.6	128	3	0.3	104	3	0.6	56	1	1.6
Multidisciplinary consultation	2198	43	0.5	1633	32	0.8	934	18	2.0	2579	45	0.4	1898	33	0.8	1068	19	1.9	1498	36	0.4	939	23	0.7	464	11	1.8
Consultation	2924	57	0.4	2139	42	0.6	1026	20	1.7	3122	55	0.4	2149	38	0.7	940	17	1.8	2128	52	0.4	1304	32	0.7	493	12	1.8
Paramedic and therapeutic care, per 15 min	2015	39	3.5	1487	29	6.4	776	15	17.8	2428	43	3.8	1838	32	7.0	931	16	18.9	1700	45	3.1	1097	29	5.8	523	14	16.8
Diagnostic tests																											
Laboratory test	4459	87	9.9	3673	72	18.0	2012	39	53.1	4984	88	10.2	3949	70	18.8	2093	37	51.7	3383	82	10.9	2315	56	21.0	1105	27	61.3
Noninvasive diagnostic test—pathological	3823	75	1.1	2942	58	2.4	1485	29	7.1	4247	75	1.1	3148	55	2.1	1562	28	6.2	2652	64	1.2	1714	42	2.4	783	19	7.9
Other noninvasive diagnostics	2651	52	0.3	1706	33	0.6	765	15	1.7	3058	54	0.3	1983	35	0.6	907	16	1.7	1954	47	0.3	1091	27	0.6	448	11	1.7
Invasive diagnostics	1907	37	0.3	1068	21	0.6	387	8	2.1	2000	35	0.3	1149	20	0.5	401	7	1.5	1260	31	0.3	628	15	0.5	192	5	1.6
Imaging																											
Conventional radiology	3388	66	0.4	2485	49	0.7	1287	25	1.9	3709	65	0.4	2618	46	0.8	1300	23	2.1	2342	57	0.4	1448	35	0.8	692	17	2.2
CT scan	3248	64	0.3	2101	41	0.6	691	14	1.6	3578	63	0.3	2276	40	0.6	758	13	1.6	2307	56	0.3	1213	29	0.6	390	10	1.6
MRI scan	686	13	0.2	390	8	0.5	97	2	1.5	794	14	0.2	409	7	0.5	102	2	1.4	457	11	0.2	191	5	0.5	35	1	1.3
PET scan	275	5	0.2	125	2	0.3	26	1	1.1	276	5	0.2	97	2	0.4	15	0	1.0	186	5	0.2	60	2	0.3	8	0	1.0
Nuclear scan other than PET	208	4	0.2	103	2	0.4	21	0	1.4	237	4	0.2	115	2	0.5	29	1	1.4	116	3	0.2	47	1	0.4	11	0	1.0
Procedures and treatments																											
Chemotherapy	1333	26	0.7	792	16	1.0	236	5	2.2	1446	25	0.7	803	14	1.0	250	4	2.2	1127	27	0.8	548	13	1.2	136	3	2.8
CRC surgery	440	9	0.2	248	5	0.4	92	2	1.4	428	8	0.3	238	4	0.5	93	2	1.4	249	6	0.3	108	3	0.5	34	1	1.6
Non-CRC surgery	1097	22	0.3	584	11	0.5	175	3	1.3	1130	20	0.3	593	10	0.5	168	3	1.4	729	18	1.3	305	7	0.5	84	2	1.7
Biologics	654	13	0.7	385	8	0.7	118	2	1.5	827	15	0.5	477	8	0.7	119	2	1.5	578	14	0.5	276	7	0.7	64	2	1.3
Radiotherapy	667	13	1.5	424	8	2.5	142	3	6.4	728	13	1.4	464	8	2.3	140	3	5.7	396	10	1.3	207	5	2.0	50	1	5.2
Other (care and items)	3653	72	1.7	2830	55	4.3	1542	30	11.8	4022	71	2.3	3049	54	4.3	1511	27	10.4	2735	66	1.5	1799	44	3.0	806	20	9.5
Lung cancer	2013 (N = 9124)									2014 (N = 9764)									2015 (N = 6643)								
Months before death	6			3			1			6			3			1			6			3			1		
	N	%	I^a^	N	%	I	N	%	I	N	%	I	N	%	I	N	%	I	N	%	I	N	%	I	N	%	I
Inpatient days																											
Hospital bed days	5978	66	1.7	4714	52	3.3	2712	30	9.0	6395	66	1.7	4936	51	3.3	2813	29	8.7	3799	57	1.7	2617	39	3.0	1421	21	8.9
ICU days	435	5	0.8	344	4	1.8	257	3	5.9	449	5	0.9	349	4	1.8	252	3	5.7	253	4	0.9	175	3	2.1	128	2	6.2
Outpatient days																											
ED visit	5200	57	0.2	3998	44	0.4	2188	24	1.2	5534	57	0.2	4136	42	0.4	2186	22	1.2	3118	47	0.2	2093	32	0.4	1076	16	1.2
Ambulatory visit	8644	95	0.5	7467	82	0.7	4209	82	0.7	9164	94	0.5	7822	20	0.7	4264	44	1.6	6153	93	0.5	4560	16	0.4	2161	33	1.7
Specialty care consultations																											
Palliative care consultation	264	3	0.3	229	3	0.5	167	2	1.5	338	4	0.2	279	3	0.5	195	2	1.5	135	2	0.3	110	2	0.6	69	1	1.7
Multidisciplinary consultation	4754	52	0.4	3616	40	0.7	2036	22	2.0	5327	55	0.4	4030	41	0.7	2283	23	1.9	3028	46	0.3	2047	31	0.6	1062	16	1.8
Consultation	551	60	0.3	4196	47	0.6	2041	22	1.4	5940	61	0.4	4436	45	0.6	2076	21	1.6	3683	55	0.4	2331	35	0.6	1014	15	1.7
Paramedic and therapeutic care, per 15 min	4043	44	2.8	3023	33	5.1	1604	18	12.7	4639	48	2.7	3502	36	4.8	1886	19	12.3	3012	45	2.5	2078	31	3.9	1073	16	11.8
Diagnostic tests																											
Laboratory test	7755	85	8.1	6317	69	15.1	3515	39	46.6	8237	84	8.7	6549	67	16.2	3559	37	48.2	5361	81	8.7	3740	56	16.3	1883	28	50.3
Noninvasive diagnostic test—pathological	7016	77	0.9	5386	59	1.7	2830	31	5.6	7492	77	1.0	5673	58	2.0	2936	30	6.1	4581	69	1.0	3045	46	2.2	1486	22	6.5
Other noninvasive diagnostics	5577	62	0.4	3775	41	0.6	1764	19	1.7	6048	62	0.4	4135	42	0.7	1875	19	1.8	3705	56	0.4	2248	34	0.6	958	14	1.8
Invasive diagnostics	4081	45	0.3	2353	26	0.6	868	10	1.6	4247	44	0.3	2408	25	0.5	887	9	1.5	2520	38	0.3	1296	20	0.5	439	7	1.5
Imaging																											
Conventional radiology	7218	79	0.4	5664	62	0.7	2906	32	2.0	7658	78	0.4	5826	60	0.8	2980	31	2.2	4779	72	0.4	3226	49	0.8	1553	23	2.3
CT scan	5920	65	0.3	3993	44	0.6	1485	16	1.6	6409	66	0.3	4280	44	0.6	1555	16	1.6	4050	61	0.3	2350	35	0.5	789	12	1.6
MRI scan	2423	27	0.2	1396	15	0.5	406	4	1.4	2600	27	0.2	1517	16	0.5	446	5	1.3	1545	23	0.2	831	13	0.5	210	3	1.4
PET scan	1795	20	0.2	808	9	0.3	170	2	1.0	2083	21	0.2	936	10	0.3	206	2	1.0	1147	21	0.2	468	10	0.3	110	2	1.0
Nuclear scan other than PET	1295	14	0.2	634	7	0.4	157	2	1.3	1422	15	0.2	658	7	0.4	157	2	1.3	832	15	0.2	364	7	0.4	90	2	1.3
Procedures and treatments																											
Chemotherapy	2724	30	0.8	1667	18	1.2	549	6	2.7	3054	31	0.7	1821	19	1.0	582	6	2.3	1969	40	0.7	983	15	0.8	260	4	2.4
LC surgery	87	1	0.2	47	1	0.4	19	0	1.4	79	1	0.2	47	1	0.4	11	0	1.1	55	1	0.2	34	0	0.3	14	0	1.0
Non-LC surgery	1355	15	0.2	689	8	0.4	199	2	1.3	1403	14	0.2	725	7	0.5	211	2	1.2	821	12	1.5	365	6	0.5	101	2	1.5
Biologics	128	1	0.4	75	1	0.7	20	0	1.2	132	1	0.4	72	1	0.6	18	0	1.5	73	1	0.4	31	1	0.5	9	0	1.5
Radiotherapy	2797	31	1.6	1898	21	2.4	707	8	5.1	2998	31	1.5	2020	21	2.5	714	7	5.7	1420	21	1.5	821	12	1.7	263	4	5.2
Other (care and items)	6181	68	1.4	4732	52	2.6	2452	27	8.5	6647	68	1.6	5050	52	2.8	2521	26	9.7	4112	62	1.1	2757	42	2.3	1292	19	7.0

Abbreviations: CRC, colorectal cancer; CT, computed tomography; ED, emergency department; I, Intensity; ICU, intensive care unit; LC, lung cancer; MRI, magnetic resonance imaging; PET, positron emission tomography.

^a^Intensity is the average number of treatments per patient per month for the patients who received a particular treatment.

In 2015, during their last 6 months of life, 55.2% of patients with CRC and 57.2% of patients with LC were admitted to hospital (not including ICU and ER admissions). In the last month of life, these proportions decreased to 18.9% and 21.4%, respectively, with an average stay of 9 hospital bed days. For patients who visited the ICU (2%-3%) and the ED (13%-16%) during their last month of life, the numbers of ICU days and ED contacts during this period were 5.5 days and 1.2 times, respectively, for patients with CRC and 6.2 days and 1.2 times for patients with LC.

Less than 2% of all patients had a palliative care team consultation in their last month of life (Table 3), while up to 20% of the patients with CRC and 23% of the patients with LC had a (multidisciplinary) consultation with their health-care specialist in this timeframe.

During the last 6 months of life, up to 66% of the patients with CRC and 79% of the patients with LC underwent conventional radiology. A total of 56% to 64% of patients with CRC and 61% to 66% for patients with LC received a computed tomography (CT) scan during this period, which dropped to 10% to 14% for CRC and 12% to 16% for LC during the last month of the patient’s life.

During their last 6 months of life, a small number of patients received cancer-related surgery, representing 9% of the patients with CRC and 1% of the patients with LC. No more than 1% of patients with LC received biologics, although 13% to 15% of patients with CRC received these treatments throughout the whole EoL phase. The opposite was true for radiotherapy, as up to 31% of the patients with LC received this treatment compared to just 13% of patients with CRC in the last 6 months of life. Overall, in the last month of life, we found that a very low percentage of patients underwent cancer-specific treatments and procedures.

### Association Between Chemotherapy/Radiotherapy and ED Contacts

Patients with CRC receiving chemotherapy had more ED contacts during their last 6 months before death than patients who did not undergo chemotherapy during this period (unadjusted odds ratio [OR] = 1.8, 95% confidence interval [CI] = 1.6-2.1; Table 4). During the last month of life, the OR increased to 17.2 (95% CI = 11.8-25.0). We observed a similar increase in patients with LC (OR = 2.0 six months before death, 95% CI = 1.8-2.2; OR = 15.8 one month before death, 95% CI = 12.0-20.9). Patients with both CRC and LC receiving radiotherapy in their last month of life were significantly more frequently admitted to the ED than patients who did not undergo radiotherapy (OR for patients with CRC = 4.6, 95% CI = 2.6-8.1; OR for patients with LC = 4.2, 95% CI = 3.2-5.4).

**Table 4.** Number of Patients With ED Contacts or ICU Days and the Number of Patients who Underwent Radiotherapy or Chemotherapy in 2015.

	Chemotherapy, N (%)	No chemotherapy, %	Crude OR [95%CI]	Radiotherapy, %	No radiotherapy, %	Crude OR [95%CI]
Colorectal cancer (N = 4123)	N = 1127	N = 2996		N = 396	N = 3727	
Last 6 months of life						
≥1 ED-contact	610 (54.1)	1174 (39.2)	1	183 (46.2)	1603 (43.0)	1
No ED-contact	517 (45.9)	1822 (60.8)	**1.8 [1.6-2.1]**	213 (53.8)	2124 (57.0)	1.1 [0.9-1.4]
≥1 ICU-day(s)	44 (3.9)	204 (6.8)	1	23 (5.8)	227 (6.1)	1
No ICU-day	1083 (96.1)	2792 (93.2)	**0.6 [0.4-0.8]**	373 (94.2)	3500 (93.9)	1.0 [0.6-1.5]
	N = 136	N = 3987		N = 50	N = 4073	
Last month of life						
≥1 ED-contact	93 (68.4)	447 (11.2)	1	20 (40.0)	517 (12.7)	1
No ED-contact	43 (31.6)	3540 (88.8)	**17.2 [11.8-25.0]**	30 (60.0)	3556 (87.3)	**4.6 [2.6-8.1]**
≥1 ICU-day(s)	6 (4.4)	116 (2.9)	1	3 (6.0)	118 (2.9)	1
No ICU-day	130 (95.6)	3871 (97.1)	1.5 [0.7-5.6]	47 (94.0)	3955 (97.1)	2.1 [0.7-6.9]
Lung cancer (N = 6643)	N = 1969	N = 4674		N = 1420	N = 5223	
Last 6 months of life						
≥1 ED-contact	1158 (58.8)	1963 (42.0)	1	728 (51.3)	2397 (45.8)	1
No ED-contact	811 (41.2)	2711 (58.0)	**2.0 [1.8-2.2]**	692 (48.7)	2836 (54.2)	**1.3 [1.1-1.4]**
≥ 1 ICU-day(s)	57 (2.9)	196 (4.2)	1	43 (3.0)	209 (4.0)	1
No ICU-day	1912 (97.1)	4478 (95.8)	**0.7 [0.5-0.9]**	1377 (97.0)	5024 (96.0)	0.7 [0.5-1.0]
	N = 260	N = 6383		N = 263	N = 6380	
Last month of life						
≥ 1 ED-contact	187 (71.9)	887 (13.9)	1	112 (42.6)	963 (15.1)	1
No ED-contact	73 (28.1)	5496 (86.1)	**15.8 [12.0-20.9]**	151 (57.4)	5417 (84.9)	**4.2 [3.2-5.4]**
≥ 1 ICU-day(s)	13 (5.0)	115 (1.8)	1	8 (3.0)	121 (1.9)	1S
No ICU-day	247 (95.0)	6281 (98.4)	**2.9 [1.6-5.2]**	255 (97.0)	6259 (98.1)	**2.7 [1.3-5.6]**

Abbreviations: CI, confidence interval; ED, emergency department; ICU, intensive care unit; OR, odds ratio.

Bold face values – Level of significance *p* < 0.05.

### Association Between Chemotherapy/Radiotherapy and ICU Contacts

Patients receiving chemotherapy 6 months before their death spent fewer days in the ICU than patients who did not undergo this treatment. We observed that, in the last month of life, patients with LC undergoing chemotherapy were admitted to the ICU (OR = 2.9, 95% CI = 1.6-5.2) more often than those who did not receive chemotherapy during this period. Patients with LC receiving radiotherapy in their last month of life were significantly more frequently admitted to the ICU than patients who did not undergo this treatment (OR = 2.7, 95% CI: = 1.3-5.6).

## Discussion

We revealed that a substantial number of patients with LC and CRC received highly intensive treatments during their EoL phase. Receiving chemotherapy or radiotherapy in the last month of life was found to be strongly associated with increased ER and ICU contacts in patients with LC.

Our results regarding hospital admissions are comparable to those of Bekelman et al who reported that 7% of elderly Dutch patients who died of cancer in 2010 spent 1 or more day(s) in ICU during their last 30 days of life, while 42% were hospitalized in an acute-care hospital.^7^ They also found that ICU admissions were more than twice as common in the United States than in the other 6 reporting countries for patients with cancer, including the Netherlands. Teno et al reported that 29.2% of all (Medicare-supported) patients with cancer in the United States were admitted to the ICU during their last 30 days of life (data from 2009).^10^ These findings were substantially higher than those of the present study (3% to 5%); however, the Netherlands has one of the lowest rates of ICU admissions among the 8 countries investigated by Teno et al.^10^ Although these findings show that HCU during the EoL phase is lower in the Netherlands than in other countries, overuse (or underuse) could still be present.

The medical experts included in this study indicated that the HCUPs we identified corresponded with their experience of daily oncological practice, describing them as “shockingly intense.” According to them, the potential areas for improvement in EoL care are to reduce the high number of admissions to the ICU and ER, to reduce the number of patients receiving a CT scan and the overall number of scans each patient receives, and to increase the number of palliative care consultations held with patients. We will discuss each of these themes and their level of appropriateness below.

Unwanted hospital admissions may be necessitated by a prolonged treatment during the EoL phase, as doctors may find it difficult to cease high-impact treatments such as chemotherapy and biological treatment in a timely manner^11^ and might not always discuss preferences and treatment aims with the patient.^12^ We argue that unplanned and even unwanted admissions could be prevented by timely discussions regarding EoL care or ACP. Palliative care consultations are a means of giving patients a more central role in their health care and the medical decisions surrounding their EoL.^13^ Although ACP has its pros and cons, recent studies have shown that ACP is able to improve the overall compliance with patient EoL wishes and improve the satisfaction of the patients and their families. It also reduces family stress, anxiety, and depression,^14-16^ while increasing the quality of life and survival rates in comparison to standard care^16^ and significantly lowering health-care costs.^6,17^ Despite these benefits, there is currently little evidence to indicate whether palliative care interventions implemented in the hospital, home, or outpatient clinic are more effective than standard care practices at reducing ED visits among patients with cancer during their EoL phase.^18^

Unfortunately, the number of patients receiving palliative care consultations in our study was exceptionally low. One explanation for this might be that, in the Dutch health-care system, the DRG data on palliative consultations are only registered as palliative care when a multidisciplinary team is actively providing palliative care, there has been at least 1 clinical or ambulant consultation, and the patient is solely receiving palliative care with no other curative treatments. A palliative consultation can be registered as a health-care activity; however, when the activity is not registered as palliative care, it is not sent to the database we used for our analyses. Although the number of palliative teams in Dutch hospitals is increasing,^19^ supportive care specialists are only involved in the care of 12% of non-sudden-death patients during their last month of life.^20^ Also, other reported data suggest that 20% to 25% of a representative sample of Dutch patients with CRC and LC had a palliative treatment aim in the 3 months before their deaths as recorded by their general practitioners (GPs).^21^ The percentage of patients who receive palliative consultations and the total number of these consultations might therefore be higher than our results suggest, although they are still likely to be relatively uncommon.

Our results suggest that high-impact treatments are associated with an increase in both days spent in the ICU in the last month of life for patients with LC and the number of ED contacts made throughout the entire EoL phase for both patients with LC and CRC. We cannot conclude that these treatments are inappropriate in the EoL phase on the basis of this study, however, as more information regarding the treatment aims and patient preferences would be required to make this determination. The association we found could reflect 2 nonexclusive contrasting possibilities: Patients receiving chemotherapy may end up in the ED/ICU more often than those who do not receive chemotherapy/radiotherapy due to an increase in complications, or their ED/ICU admission may lead to chemotherapy treatments; for instance, if a palliative treatment aim is determined after their admission. Studies have shown conflicting and inconclusive results regarding (palliative) chemotherapy^22-24^; however, regardless of the direction of this association, our data suggest that high-intensity care (whether chemotherapy or an ICU admission) is associated with the patient receiving other highly intensive care. Medical specialists should consider the intensity of these treatments when discussing them with their patients. Bearing in mind that 80% of patients would prefer to die at home^25,26^ but only 61% actually do,^26^ we must ask whether this trend is acceptable.

Although they are important for the initial staging process of the cancer with acceptable cost-effectiveness parameters and even cost savings,^27^ a CT scan might be unnecessary if it will not make a difference to the subsequent treatment. Schnipper et al even stated that “until high-level evidence demonstrates that routine surveillance with PET or PET/CT scans helps prolong life or promote wellbeing after treatment for a specific type of cancer, this practice should not be performed.”^28(p4365)^ Given this recommendation, it is remarkable that an average of 1.6 CT scans were still performed on almost 10% of patients in their final month of life or an average of 0.6 times for almost 30% of patients during their final 3 months.

### Strengths and Limitations

A major strength of our study was the analysis of the data at the level of health-care activities. Although our database only included hospital data, the use of these data enabled us to analyze HCU in detail. Furthermore, the involvement of a panel of medical experts enabled us to gather specialized input and opinions and subsequently understand and interpret the results. Our data were shown to be representative, as the mean age at death and the male–female ratio for both cancer types were comparable to the national mortality data.^1^

Our study also had some limitations, such as the fact that we were not able to distinguish between curative and palliative treatments in these data. This is important, as it would give more insight into possible explanations for the HCU we have found in our study. Also, registered health care does not always necessarily reflect the actual health care supplied; faulty registrations or nonregistrations do occur. Furthermore, the 2015 database was not complete at the time of this study due to the time-consuming process of collecting data from Dutch hospitals. When comparing the data from each year, however, we found that our results were robust. We therefore argue that the incompleteness of the data did not substantially alter the image of the HCUP.

Although high-intensity hospital care is given to patients in the EoL phase, some patients received no hospital-based care. We were unable to investigate the differences in characteristics and HCU between these 2 groups, but we believe that the hospital-based patients are most likely to receive high-intensity and high-cost, maybe even inappropriate, care during the EoL phase in comparison to the nonhospital-based patients. We do not know whether the care for nonhospital-based patients was more appropriate than the hospital care provided to other patients. In order to further investigate this, any geographical or institutional variations in HCU within the Netherlands need to be identified, as these are even stronger indications of potential overuse and underuse. We also need to broaden the scope of future studies by analyzing the full health-care chain to determine which types of primary care were provided for patients with high levels of secondary care utilization and to identify the underlying reasons for differences in HCU. The ultimate aim would be to signal the overuse and underuse of health care while also understanding it, leading to better conversations between physicians/institutions and patients to improve quality of care.

In conclusion, we observed a high usage of intense care in the EoL phase of patients with CRC and LC in the Netherlands, which may represent the overuse of health care, especially regarding hospital admissions and CT scans. Palliative care needs to be further improved and developed in the EoL phase and may currently be underused. Advanced care planning can play a substantial role in ensuring the appropriate use of palliative care. More research is necessary to identify the true overuse and underuse of hospital medical services.
